# Nicorandil reduces the antimicrobial effectiveness of polymyxin E against *Klebsiella pneumoniae* by decreasing reactive oxygen species accumulation

**DOI:** 10.3389/fcimb.2025.1658194

**Published:** 2025-09-17

**Authors:** Rongqing Zhu, Fangxin Luo, Chenxi Yuan, Ziqian Fang, Yaqin Guo, Bao Meng, Dongmei Zhao, Yanyan Liu, Yi Yang, Yasheng Li, Jiabin Li, Liang Yu

**Affiliations:** ^1^ Department of Infectious Disease & Anhui Center for Surveillance of Bacterial Resistance, The First Affiliated Hospital, Anhui Medical University, Hefei, China; ^2^ Anhui Province Key Laboratory of Infectious Diseases, The First Affiliated Hospital of Anhui Medical University, Hefei, China; ^3^ Institute of Bacterial Resistance, Anhui Medical University, Hefei, China

**Keywords:** nicorandil, polymyxin E, *Klebsiella pneumoniae*, reactive oxygen species, nitric oxide

## Abstract

Nitric oxide (NO) plays a crucial role in bacterial physiology and survival, particularly in relation to antibiotic resistance. The protective role of NO against antibiotics is intricate, and the potential antagonistic interactions between NO donors and polymyxin E remain largely unexplored. This study aimed to evaluate the antagonistic effects of nicorandil, a NO donor, on the bactericidal activity of polymyxin E against *Klebsiella pneumoniae*. Methods: Thirty clinical strains were identified as multidrug-resistant *K. pneumoniae* using matrix-assisted laser desorption/ionization time-of-flight mass spectrometry (MALDI-TOF MS). The antimicrobial efficacy of polymyxin E combined with nicorandil against *K. pneumoniae* was evaluated through *in vitro* rapid killing assays and growth curve analyses, and *in vivo* using a murine pulmonary infection model and a *Galleria mellonella* larvae infection model. The release of NO by nicorandil was confirmed via reactive nitrogen species (RNS) assays. The impact of NO on oxidative stress responses induced by polymyxin E was evaluated using reactive oxygen species (ROS) assays and RT-qPCR. Results: Nicorandil counteracted the bactericidal effects of polymyxin E in 16 out of 30 clinical isolates of *K. pneumoniae*. Notably, the most pronounced effects were observed in the *K. pneumoniae* strain GN 191035. In this context, the release of NO from nicorandil conferred protection to the bacteria against oxidative stress by reducing ROS, as demonstrated by a murine model of pulmonary infection and a *Galleria mellonella* larvae infection model. Conclusions: Our study further elucidated that nicorandil treatment mitigates the bactericidal efficacy of polymyxin E against *K. pneumoniae*. These findings highlight the significant risk of increased bacterial infections associated with the concurrent administration of nicorandil and polymyxin E.

## 1 Introduction

Polymyxin E, also known as colistin, is a crucial last-resort treatment for infections caused by multidrug-resistant Gram-negative bacteria (Mendelson et al., 2018). This development is attributed to the escalating incidence of carbapenem-resistant organisms, which significantly constrain the therapeutic options accessible to healthcare professionals (Hu et al., 2024). In recent years, the extensive use of polymyxin E for treating multisystem infections induced by carbapenem-resistant (Huang et al., 2024) and multidrug-resistant *Klebsiella pneumoniae* has resulted in reduced therapeutic effectiveness (Band et al., 2018). Consequently, this has facilitated the emergence of polymyxin E-resistant gram-negative pathogens.

Bactericidal antibiotics, such as polymyxins, generate reactive oxygen species (ROS), leading to cell death (Mi et al., 2016; Sampson et al., 2012). Nitric oxide, generated by bacterial nitric oxide synthase (bNOS) or from the donor MAHMA NONOate, increases bacterial antibiotic resistance, aiding in their survival (Gusarov et al., 2009). The mechanism of NO-mediated resistance involves the mitigation of oxidative stress caused by numerous antibiotics, suggesting that inhibiting bNOS activity could potentially enhance the efficacy of antimicrobial therapies (Gusarov and Nudler, 2005). Prior studies have shown that a few bNOS inhibitors can enhance the effectiveness of antimicrobial agents (Holden et al., 2015).

As the development of resistance can be attributed, in part, to the antagonism that may occur with concurrent medication regimens (Ocampo et al., 2014). The mechanisms underlying antagonism involve mitigating the oxidative stress response, as observed with agents such as doxofylline (Chen et al., 2021) and curcumin (Marathe et al., 2013). These agents reduce the levels of ROS induced by antimicrobial agents, consequently diminishing the bactericidal efficacy of antibiotics. These results highlight the need for caution when using antibiotics in conjunction with other drugs for clinical treatment. Research indicates that NO enhances bacterial resistance to various antibiotics, such as β-lactam antibiotics (Jones-Carson et al., 2014) and aminoglycosides (McCollister et al., 2011; Zemke et al., 2015). Some studies have proposed that NO-mediated resistance is facilitated by activated catalase, thereby affecting oxidative stress (Gusarov and Nudler, 2005, 2009). Polymyxin E is considered the last resort for treating infections by multidrug-resistant *K. pneumoniae*. Nonetheless, there is presently a lack of research investigating whether NO contributes to increased bacterial resistance to polymyxin E.

Nicorandil, known for the nitrate group (-ONO_2_) in its molecular structure, facilitates the release of NO, which aids vasodilation and pain relief (Kotoda et al., 2018). Nicorandil’s antioxidant properties stem from its capacity to directly scavenge hydroxyl radicals, thereby inhibiting free radical production. It regulates NO homeostasis and exerts antioxidant effects that reduce inflammatory and apoptotic responses (Serizawa et al., 2011; Yamanaka et al., 2023; Zhang et al., 2015). It is commonly prescribed for elderly individuals with ischaemic heart disease. Antimicrobials like polymyxin E, which cause oxidative stress leading to bacterial cell death, are used to treat infections by multidrug-resistant (MDR) bacteria (Lima et al., 2019; Sampson et al., 2012). Nonetheless, the interaction between the antioxidant properties of nicorandil and the mechanisms of action of antimicrobial agents, especially in the context of Carbapenem-Resistant Enterobacterales infections, remains inadequately understood.

Therefore, this study aimed to evaluate the activity of nicorandil in combination with polymyxin E against *K. pneumoniae*. The study partially elucidated the molecular mechanism through which nicorandil influences the resistance of *K. pneumoniae* to polymyxin E.

## 2 Materials and methods

### 2.1 Strains and reagents

Thirty clinical *K. pneumoniae* strains were isolated from respiratory samples at the Anhui Center for Surveillance of Bacterial Resistance (Liu et al., 2022). Identification of all strains as *K. pneumoniae* was confirmed using a MALDI-TOF MS automated microbiology system (BIOYONG, Beijing, China). Isolates were preserved in cryovials containing Muller-Hinton broth (MHB; Sigma-Aldrich, St. Louis, MO, USA) with 50% glycerol at − 80°C and cultured on Muller-Hinton agar (MHA; Sigma-Aldrich, St. Louis, MO, USA) at 37°C. Antibiotics, nicorandil, N-Ethylnicotinamide, and N-(2-Hydroxyethyl) nicotinamide were sourced from Sigma-Aldrich (St. Louis, MO, USA). Solvents and diluents used for antibiotic preparation adhered to the latest Clinical and Laboratory Standards Institute guidelines (CLSI, 2020). Eight-week-old wild-type female C57BL/6 mice, weighing 16 to 20 g, were obtained from the Experimental Animal Center in Anhui Province, Hefei, China. Final instar *G. mellonella* larvae (Kaide ruixin Co., Ltd, Tianjin, China) were kept in the dark at 4°C and utilized within 7 days of receipt. Larvae masses were generally around 250 mg, which was used to determine treatment doses. The Institutional Animal Care and Use Committee of Anhui Medical University approved all experiments involving mice (approval no. LLSC20190253).

### 2.2 Antimicrobial susceptibility testing

The MIC of all antibiotics against *K. pneumoniae* was assessed via the broth microdilution method, following EUCAST and CLSI guidelines. After overnight culture, bacterial concentration was set to 0.5 McFarland standard in sterile saline and then diluted 1:100 in MHB. Subsequently, all drugs were diluted twofold in MHB broth and combined with bacterial suspensions in a 96-well microtiter plate. MIC values were determined as the minimum drug concentrations that inhibited visible bacterial growth after 18 hours of incubation at 37°C.The lowest concentration of polymyxin E which inhibits visible bacteria growth in turbidity form was recognized as its MIC value. MIC of colistin was interpreted in accordance with the CLSI breakpoints, with resistance defined as a MIC of ≥ 4 mg/L. Wells containing bacterial cells served as positive controls, while those without were used as negative controls (Chen et al., 2022; Yi et al., 2022).

### 2.3 Rapid killing assay

Rapid killing assay of 30 clinical *K. pneumoniae* strains and one standard strain ATCC 43816, which were determined during the exponential growth phase. The strains were cultured in 5 mL of MHB broth and incubated overnight at 37°C with shaking at 220 rpm. The amplified bacterial cultures were diluted to a turbidity equivalent to 0.5 McFarland standard using Mueller-Hinton Broth to achieve a final volume of 5 mL. The diluted bacterial suspensions were then incubated with varying concentrations of polymyxin E, nicorandil, and a combination of both agents. Incubation was conducted at 37°C with agitation at 220 rpm. Samples were collected at 0, 2 and 4 hours, and 200 μL of each sample was diluted 10–10^6^ times with 1 × PBS. Labeled MHA agar plates were inoculated with 10 μL of diluted bacterial suspensions. Bacterial colonies were counted after an incubation of 12 h to 14 h at 37°C. The antagonistic activity was identified as a reduction of ≥ 2 log_10_ when comparing the combination of two medications to each drug individually (Zhou et al., 2022). Untreated control was included in each experiment.

### 2.4 Growth curve determination

Select strains were cultured overnight in 5 mL of MHB medium at 37 °C with shaking at 220 rpm. The amplified bacteria were diluted to 0.5 McFarland turbidity using MHB broth in saline, and then further diluted 1:100 with MHB broth to a final volume of 5 mL. Diluted bacteria were treated with varying concentrations of polymyxin E, nicorandil, and their combination. A 100 μL aliquot of bacterial culture was dispensed into each well of a 96-well flat-bottom microtiter plate (Costar). Each condition was tested in quadruplicate. Cultures were grown in a plate reader (Tecan, Männedorf, Switzerland) at 37 °C until reaching the stationary phase, with the optical density (OD) value at 600 nm UV taken every 60 minutes for a total of 24 hours (Bray et al., 2022; Zhou et al., 2022).

### 2.5 Reactive oxygen species production

To detect intracellular ROS accumulation (Guo et al., 2023), we used the fluorescent probes carboxy-H2DCFDA (Beyotime, Shanghai, China). GN 191035 bacteria overnight cultures were diluted 1:100 using MHB broth. Experiments were conducted in 50 ml Eppendorf tubes with treatments of nicorandil (64 μg/mL), sub-MIC polymyxin E (32 μg/mL), and their combination. Optical density at 600 nm (OD600) was measured for each group after 4 hours. A 200 μl aliquot of bacterial culture from each group was transferred into a 1.5 mL EP tube. After a Centrifugation at 12,000 rpm for 5 minutes, the supernatant was discarded. The remaining MHB broth was washed off using 1 × PBS for 3 times. Both treated and untreated samples were incubated with carboxy-H2DCFDA at a final concentration of 10 μM for 30 minutes. Before the detection of intracellular ROS, samples were centrifuged, and washed twice with pre-cooled 1 × PBS to remove the remaining reagents. A BD FACSCelesta flow cytometer (Franklin Lakes, NJ, USA) was utilized to assess fluorescence intensity (X-axis) and cell count (Y-axis), indicating ROS production. 100,000 bacteria were included in one time. Data were analyzed using FlowJo version 10.8.1. The results are presented as the mean ± S.D. from three technical and three biological replicates.

### 2.6 Reactive nitrogen species production

To detect intracellular RNS accumulation (Gonzalez-Magallanes et al., 2023), we used the DAF-FM DA (Beyotime, Shanghai, China). *K. pneumoniae* GN 191035 in exponentially growing stage were incubated with DAF-FM DA at 5 μM for 30 minutes. Samples were washed twice with pre-cooled 1 × PBS to remove remaining reagents, followed by nicorandil, N-Ethylnicotinamide, N-(2-hydroxyethyl) nicotinamide (0 μg/mL, 64 μg/mL) treatment. After 120 minutes, treatment was ended. 200 μL sample was washed twice with pre-cooled 1 × PBS to remove the reagents. The fluorescence intensity and cell count, indicative of RNS production, were measured using a BD FACSCelesta flow cytometer (Franklin Lakes, NJ, USA). 100,000 bacteria were included in one time. Data were analyzed using FlowJo version 10.8.1. The results are presented as the mean ± S.D. from three technical and three biological replicates.

### 2.7 RNA extraction

Bacterial strains were cultured in 5 mL of MHB medium at 37°C with 220 rpm shaking overnight. Bacteria cultured overnight were diluted in saline with MHB broth to achieve 0.5 McFarland turbidity, then further diluted 1:100 in MHB broth to a final volume of 5 mL. Diluted bacteria were incubated with nicorandil (64 μg/mL), sub-MIC polymyxin E (32 μg/mL), and their combination. After 4 hours, OD600 was measured for each group. A 1 ml aliquot of the bacterial suspension was transferred to a 1.5 ml RNase-free EP tube. Samples were centrifuged at 12,000 rpm and 4°C for 5 minutes. After discarding the supernatant, 1 ml of RNAiso Plus (TaKaRa, Kyoto, Japan) was added, and the suspension was pipetted thoroughly to ensure complete bacterial lysis. The mixture was left at room temperature for 20 minutes. Subsequently, 200 μL of chloroform was added to each tube, inverted to mix, and incubated for 5 minutes at 4°C. Samples underwent centrifugation at 12,000 rpm and 4°C for 10 minutes, achieving phase separation. The upper aqueous phase was transferred to a new 1.5 mL RNase-free Eppendorf tube, and an equal volume of isopropanol was added. The mixture was incubated at room temperature for 20 minutes, followed by centrifugation at 12,000 rpm and 4°C for 10 minutes to precipitate RNA. The supernatant was discarded, and 500 μL of anhydrous ethanol was added, with gentle mixing. Samples were centrifuged under the same conditions, and the ethanol wash was repeated three times. After ethanol evaporation, the RNA pellet was dissolved in 10-50 μl of DEPC-H_2_O (TaKaRa, Kyoto, Japan). NanoDrop One spectrophotometer (Thermo, Shanghai, China) was used to measure RNA concentration and purity (Lu et al., 2020).

### 2.8 Analysis using quantitative real-time PCR

Reverse transcription was conducted using the measured RNA concentration. The reaction mixture (10 μL) contained 500 ng of total RNA, 2 μL of 5 × RT PCR Mix (TaKaRa, Kyoto, Japan), and RNase-free DEPC-H_2_O to bring the total volume to 10 μL. The reaction proceeded under the following thermal cycling conditions: incubation at 37°C for 15 minutes, followed by 85°C for 5 seconds, and finally holding at 4°C indefinitely. The cDNA generated from the reverse transcription was subsequently used for real-time quantitative PCR (qPCR). The qPCR reaction mixtures (20 μL) comprised 10 μL of TB Green premix Ex Taq II (TaKaRa, Kyoto, Japan), 0.5 μL each of forward and reverse primers, 1 μL synthesized cDNA, and double-distilled water (ddH_2_O) to reach a final volume of 20 μL. Quantification cycling was performed on a LightCycler 96 real-time PCR system (Roche, Basel, Switzerland) with an initial denaturation at 95°C for 30 seconds, followed by denaturation at 90°C for 5 seconds, annealing at 60°C for 30 seconds, and extension at 72°C for 20 seconds. All steps were repeated for 40 cycles. DNA and cDNA were quantified against a standard curve using genomic DNA and qPCR products. RT-qPCR involving *sodA*, *sodC*, *katE*, and *katG* was performed. The *rrsE6* amplicon served as an internal control. All procedures were carried out under light-protected conditions to prevent any potential photodegradation of reagents. The primers for RT-qPCR are listed in **Supplementary Table S2**. The expression levels of the target genes of interest were determined by the 2^-ΔΔCT^ calculation method and reported as fold change values. Each reaction was conducted three times.

### 2.9 Murine lung infection model

Building on prior research (Tian et al., 2024; Wu et al., 2020), we developed lung infection models to assess the effect of nicorandil on bacterial activity *in vivo*. Female C57BL/6 mice (8 per group) were anesthetized using sodium pentobarbital. A lung infection was induced by administering 50 μL of an early log-phase bacterial suspension (GN 191035, 1.5×10^7^ CFU/mL). Bacteria were administered directly into the lungs through the trachea using a nasal device. Nicorandil was prepared in sterile saline. Four hours post-inoculation, a cohort of mice (n = 8) received intraperitoneal injections of either polymyxin E at a dosage of 5 mg/kg/day, nicorandil at dosages of 20 mg/kg/day, a combination of both agents, or phosphate-buffered saline. These treatments were administered in two divided doses every 8 hours. Subsequently, prior to the administration of polymyxin E, nicorandil, their combination, or phosphate-buffered saline (serving as the control at 0 hours), and at 24 hours following the administration of these treatments, the mice were euthanized to facilitate the collection of lung tissue samples. Right lung is for pathological examination, left lung lobes were grinded. 1000 μL grinded lung lobes was serially diluted in phosphate buffered saline, and spread on nutrient agar plates. Agar plates were incubated at 37°C overnight for bacterial colony counting and the bacterial load of the lung (log_10_ CFU/lung) in each mice was calculated. *in vivo* efficacy was determined by calculating the change in log_10_ CFU per lung, comparing the treated mouse at 24 hours to the control at 0 hours (Δlog_10_ CFU/lung = log_10_ [treated] CFU/lung − log_10_ [control] CFU/lung). For histological analysis, lung tissues were fixed with paraformaldehyde, sectioned into 4-mm slices, deparaffinized, and stained using hematoxylin and eosin (H&E). Pathology scoring followed a previously established method. The total lung inflammation score was calculated by summing the scores for each parameter, with a maximum possible score of 24 (Roberts et al., 2022; Schouten et al., 2014).

### 2.10 Larvae of the wax moth (*Galleria mellonella*) infection model

The efficacy of polymyxin E, both individually and combined with *K. pneumoniae*, was assessed using modified survival assays on *G. mellonella* infected with GNB, as previously described (Cutuli et al., 2019; Yang et al., 2017). The experimental studies utilized larvae that were 5 weeks old. Larvae measured 2 to 2.5 cm in length and exhibited a creamy color. Larvae were stored at 4°C before been used, and they were starved for 24 h before infection. The primary infection route is intrahemocoelic injection via the last left pro-leg or skin. Each experimental trial utilized 16 larvae. After infection, the larvae was maintained at temperatures up to 37°C. The overnight cultures of *K. pneumoniae* (GN 191035) were adjusted to 1×10^5^ CFU/mL with sterile NS. Larvae injected with standard saline acted as controls. Sixteen larvae per group received a 10 μL bacterial suspension injection into the left hind limb using a microsyringe. Treatments administered 2 hours post-infection included polymyxin E (0.01 μg, 0.02 μg), nicorandil (0.64 μg), a combination of polymyxin E and nicorandil, or a control treatment with phosphate-buffered saline. Larvae were incubated aerobically at 37°C for three days, with *G. mellonella* survival rates recorded every 12 hours. Each experiment was conducted in triplicate. Larvae were considered dead if they showed no response to repeated physical stimuli.

### 2.11 Statistical analysis

**Figure 1:**
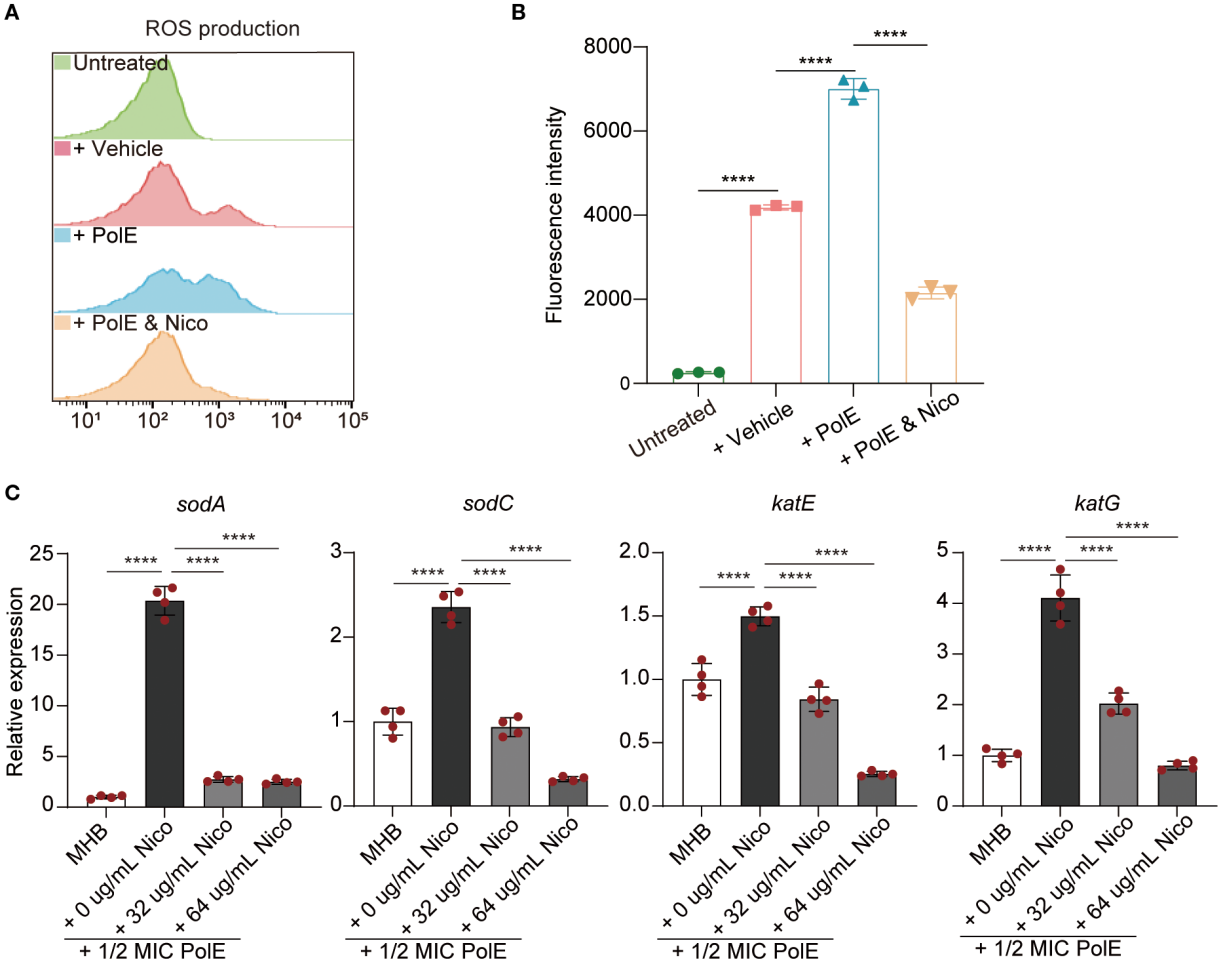
Nicorandil-treatment decreased reactive oxygen species (ROS) accumulation, leading to the protection of bacterial cells. **(A, B)** *K*. *pneumoniae* GN 191035 samples were subjected to four different treatments: untreated, vehicle-treated, polymyxin E-treated, and polymyxin E with nicorandil-treated for 4 hours. Subsequently, all samples were incubated with 10 mM carboxy-H2DCFDA for 30 minutes. Flow cytometry was utilized to measure ROS levels. Data are expressed as mean ± standard deviation (n = 3). **(C)** Real-time quantitative PCR. The mRNA expression levels of *sodA*, *sodC*, *katE*, and *katG* were evaluated in the control group, the polymyxin E-only group, and the group treated with a combination of polymyxin E and nicorandil. The mRNA expression levels were normalized relative to *rrsE6*. Data are expressed as mean ± standard deviation (n = 4).

**Figure 2:**
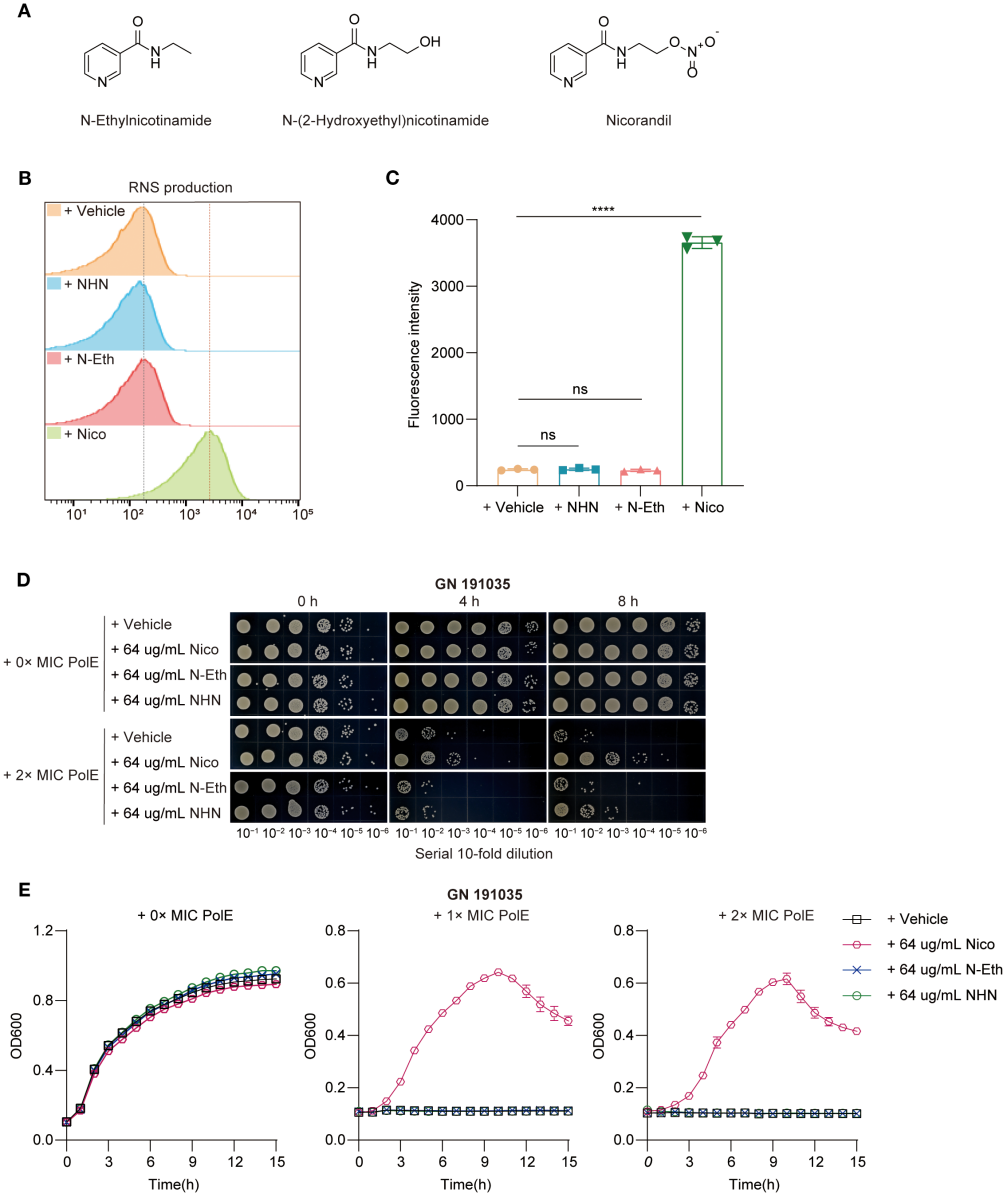
Nicorandil-treatment release NO, leading to the protection of bacterial cells. **(A)** The structure of nicorandil and two structural analogues lacking the nitrate group, N-ethyl nicotinamide and N-(2-hydroxyethyl) nicotinamide. **(B, C)** Reactive nitrogen species (RNS) production. Clinical *K. pneumoniae* GN 191035 samples were exposed to either a vehicle control, N-(2-hydroxyethyl) nicotinamide, N-Ethylnicotinamide, or nicorandil for 4 hours, followed by a 30-minute incubation with DAF-FM DA. Flow cytometry was employed to measure RNS levels. Data are expressed as mean ± standard deviation (n = 3). **(D)** Rapid killing assay. GN 191035 were treated with polymyxin E and nicorandil, N-Ethylnicotinamide, N-(2-hydroxyethyl) nicotinamide, either alone or in combination. Samples were collected at different time points and subjected to a 10-fold serial dilution. Experiments were conducted a minimum of three times, with representative outcomes reported. **(E)** Bacterial growth curve assay. GN 191035 was cultured in 96-well plates with MHB medium, supplemented with specified concentrations of polymyxin E, nicorandil, N-ethylnicotinamide, and N-(2-hydroxyethyl) nicotinamide. Data are expressed as mean ± standard deviation with a sample size of four.

**Figure 3:**
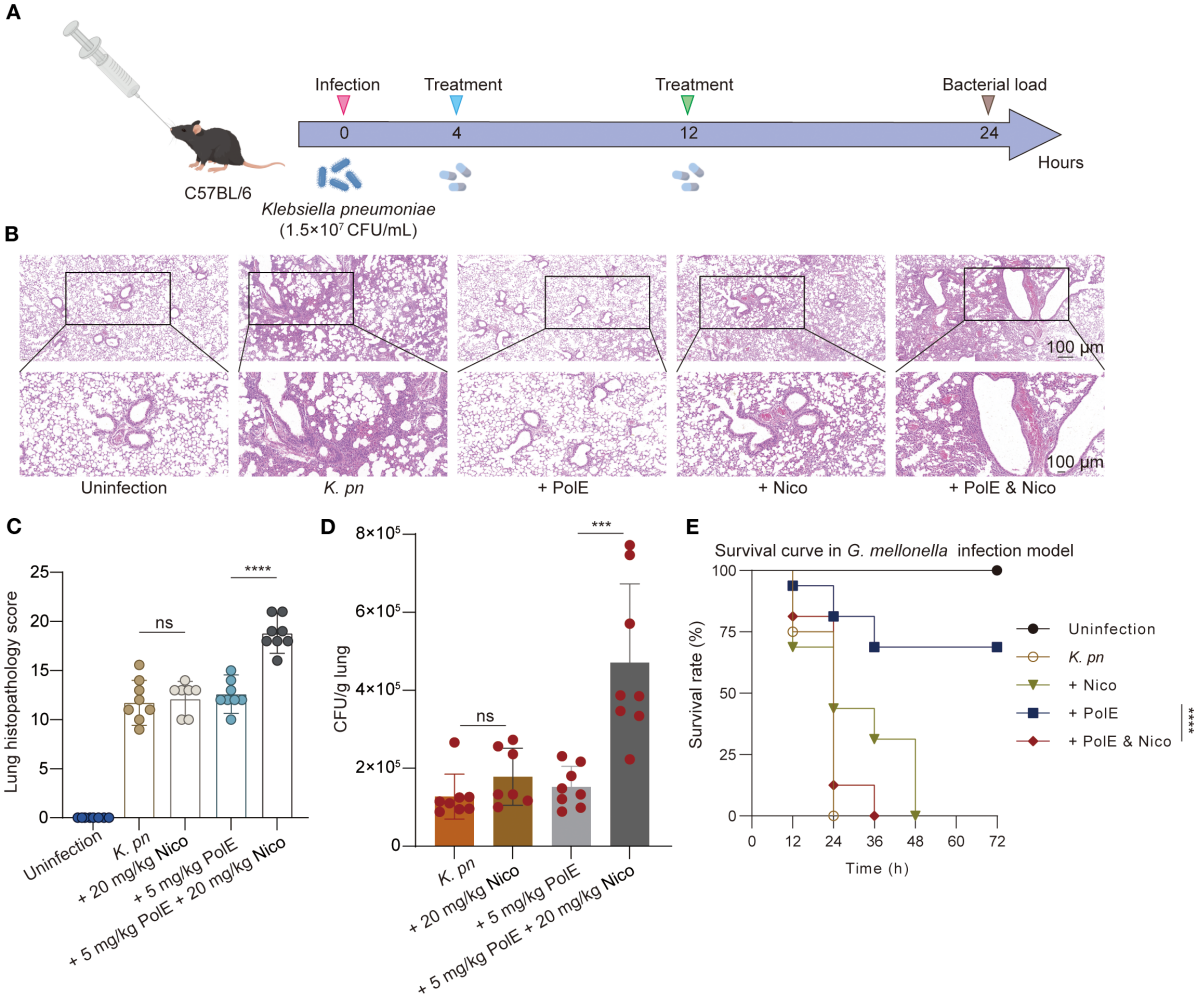
Polymyxin E and nicorandil: their impact on injury induced by *K*. *pneumoniae* GN 191035. **(A)** Schematic illustration of experimental protocol for the mice trial. C57BL/6 mice were injected with 1.5×10^7^ CFU of *K*. *pneumoniae* GN191035. The treatment was administered twice, with each group consisting of 8 mice. The CFU of *K*. *pneumoniae* in lung tissues of infected mice was measured 24 hours post-infection following treatment with nicorandil (20 mg/kg), polymyxin E (5 mg/kg), or their combination. **(B)** Representative lung slides of cells post *K*. *pneumoniae* infection, comparing untreated and antibiotic-treated groups; scale bar = 100 μm (10×). The image exemplifies findings from three separate experiments. **(C)** Pathology scoring illustration. Histopathological scores of lung tissues for each group. **(D)** Bacterial load in the lungs of infected mice with different treatments. **(E)** Survival of *G*. *mellonella* larvae post-inoculation with GN 191035. Larvae were infected with *K*. *pneumoniae* GN 191035 and left untreated, treated with polymyxin E alone, nicorandil alone, or a combination of both. Each treatment included 16 larvae. The plotted data represent the averages of four biological replicates, with error bars indicating the standard deviation. Statistical significance is denoted as follows: * for P < 0.05, ** for P < 0.01, *** for P < 0.001, and **** for P < 0.0001.

The data are averages from three separate experiments, with error bars indicating the standard errors. Group comparisons in **Figures 1B, C**, **2C**, **3C, D** were performed using Student’s two-tailed unpaired t-test. Additionally, the log-rank (Mantel-Cox) test was employed to compare overall survival between groups, as illustrated in **Figure 3E**. Statistical significance is denoted as follows: * for *P* < 0.05, ** for *P* < 0.01, *** for *P* < 0.001, and **** for *P* < 0.0001.Graphs were created using Prism 10.0 (GraphPad, Inc., San Diego, CA, USA), FlowJo 10.8.1 (Ashland, OR, USA), and Illustrator CC 2024 (Adobe Systems, Inc., USA).

## 3 Results

### 3.1 Clinical isolates of multidrug-resistant *K. pneumoniae*

**Figure 4:**
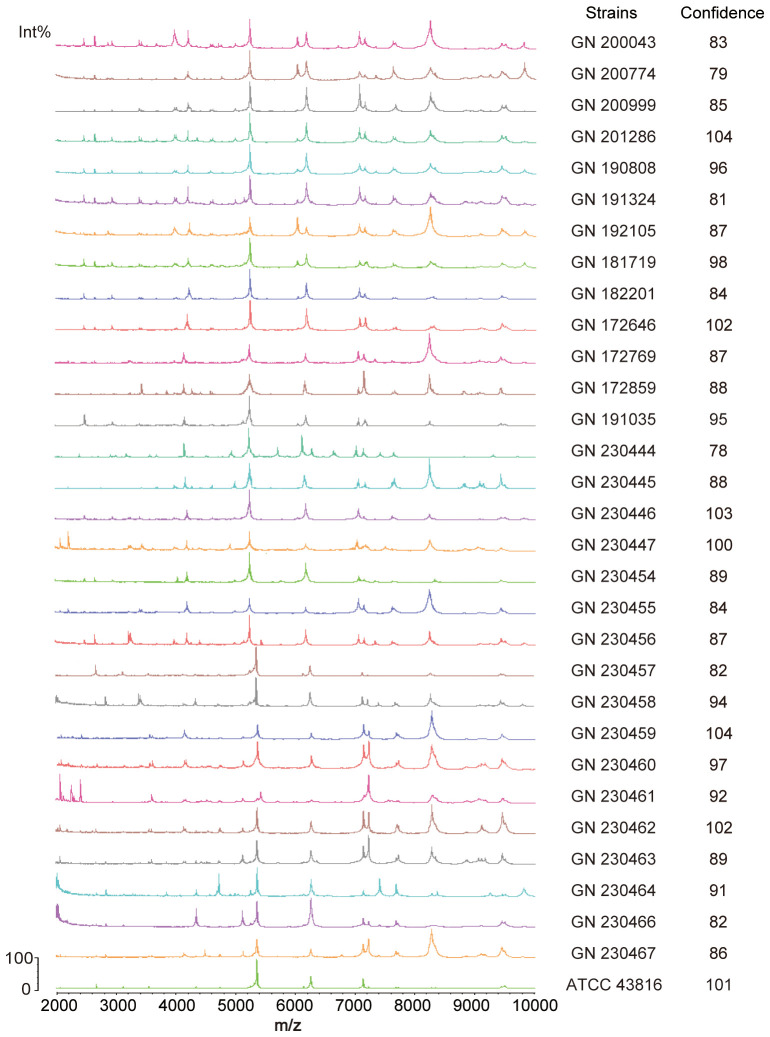
Identification of 30 clinical *Klebsiella pneumoniae* strains by MALDI-TOF mass spectrometry. The measurement range shown in the figure is from 2000 to 10000 Da, with characteristic mass peaks primarily representing ribosomal proteins. The characteristic mass peaks of *K. pneumoniae* are 4365, 5381, and 7158, with a deviation of within 5 considered normal. The computer automatically compares the generated spectra with the MALDI-TOF database and displays identification results, indicating similarity to reference spectra through a score. An identification result with a score greater than 25 is considered reliable.

This study involved 30 clinical strains of *K. pneumoniae* sourced from the Anhui Center for Surveillance of Bacterial Resistance. All strains were identified using an automated MALDI-TOF MS microbiology system (**Figure 4**). The broth microdilution method was used to evaluate the minimum inhibitory concentrations (MICs) of 11 antibiotics. All (100%) *K. pneumoniae* isolates (30/30) were identified as carbapenem-resistant MDR *K. pneumoniae* strains, whereas 20% (6/30) of the isolates demonstrated resistance to polymyxin E (**Supplementary Table S1**).

### 3.2 Effect of nicorandil on the antibacterial activity of polymyxin E

This study explored the interactions between nicorandil and polymyxin E, focusing on nicorandil’s impact on the antimicrobial efficacy of polymyxin E against *K. pneumoniae*. A rapid killing assay was performed using 30 clinical isolates and the ATCC 43816 strain. When nicorandil was co-administered with polymyxin E, an antagonistic interaction was observed in 51.6% (16 out of 31) of the isolates, leading to an increase of 1–3 log_10_ CFU/mL in the growth of *K. pneumoniae* (**Supplementary Figures S1**, **S2**, and **S3**). Conversely, an additive effect was detected in 48.4% (15 out of 31) of the isolates (**Supplementary Figures S4**, **S5**).

**Figure 5:**
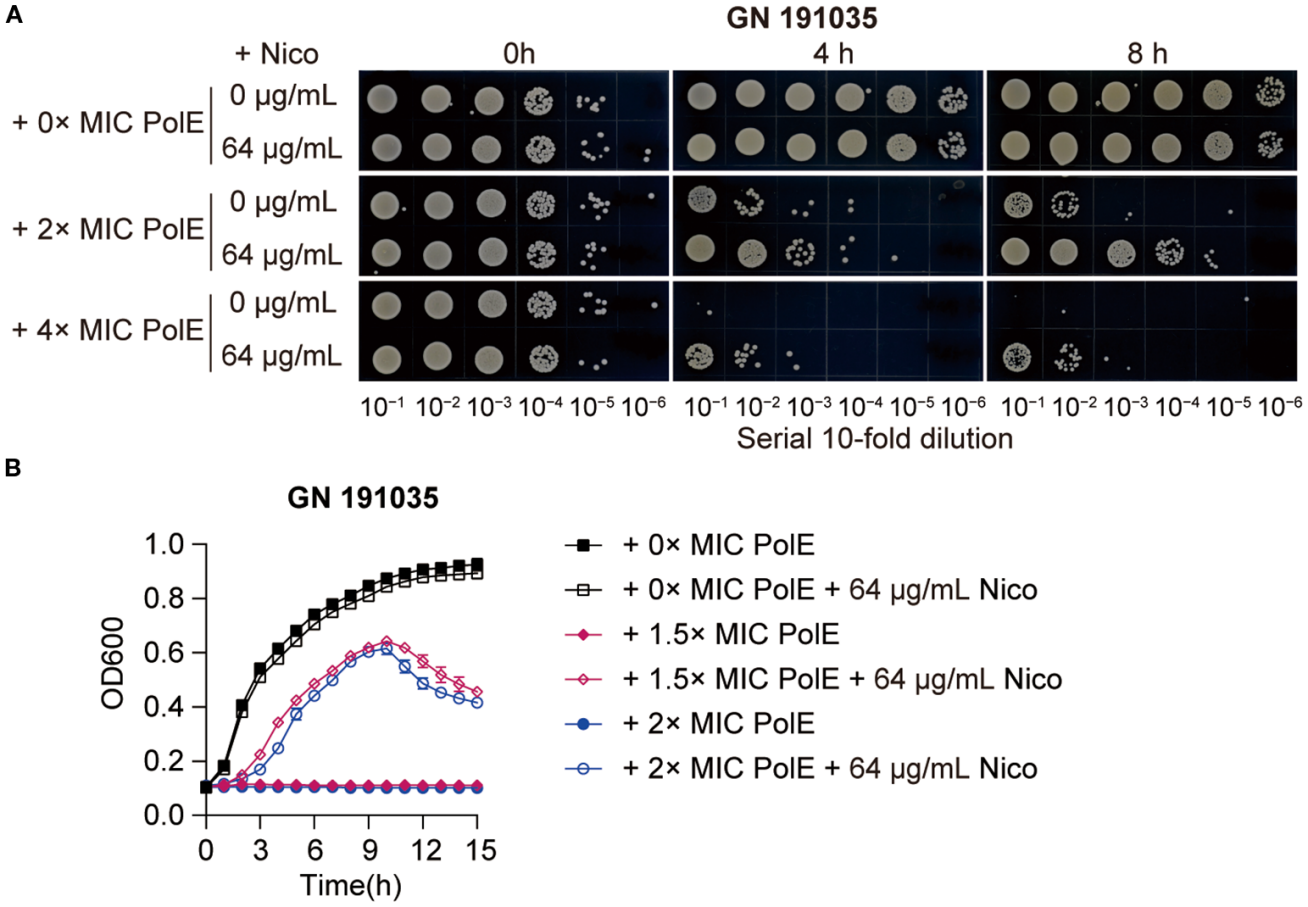
Effect of polymyxin E in combination with nicorandil on *K*. *pneumoniae*. **(A)** Rapid killing assay. The GN 191035 strain was treated with polymyxin E and nicorandil, either alone or in combination. Samples were collected at different time points and subjected to a 10-fold serial dilution. The diluted bacterial suspensions (10 µL) were spotted on MHA agar plates, and the plates were incubated at 37°C for 12 h before being photographed. Experiments were conducted a minimum of three times, with representative results shown. **(B)** Bacterial Growth Curve Assay *K. pneumoniae* strain GN 191035 was cultured in 96-well plates with MHB medium, supplemented with specified concentrations of polymyxin E and nicorandil. Results are expressed as mean ± standard deviation (n = 4).

Notably, the effects observed with the *K. pneumoniae* strain GN 191035 were the most pronounced. The administration of 64 µg/mL nicorandil alone did not influence bacterial growth. However, the combination of 128 µg/mL or 256 µg/mL (2 × MIC and 4 × MIC) polymyxin E with 64 µg/mL nicorandil significantly diminished the bactericidal activity, resulting in a comparable increase of 2 to 3 log_10_ CFU/mL (**Figure 5A**). To investigate the effect of nicorandil on the antimicrobial efficacy of polymyxin E against GN 191035 cells, a growth curve assay was conducted. The assay involved the administration of varying concentrations of polymyxin E (0 ×, 1.5 ×, and 2 × MIC) in combination with nicorandil. The results demonstrated that polymyxin E alone effectively eradicated bacteria; however, when combined with nicorandil, notable and rapid bacterial proliferation was observed (**Figure 5B**). These findings suggest that nicorandil could diminish the antimicrobial effectiveness of polymyxin E against specific bacterial strains.

### 3.3 Nicorandil mitigates the oxidative stress induced by polymyxin E

Research has shown that polymyxin E triggers hydroxyl radical production in gram-negative bacteria, leading to cell death (Lima et al., 2019; Sampson et al., 2012). The effect of nicorandil on polymyxin E-induced ROS accumulation was assessed by measuring intracellular ROS levels using carboxy-2’,7’-dichlorodihydrofluorescein diacetate (carboxy-H2DCFDA). Flow cytometric analysis revealed elevated ROS levels in the polymyxin E, whereas the nicorandil-treated group did not exhibit significant alterations (**Figures 1A, B**). Notably, the combined treatment with *K. pneumoniae* demonstrated a reduction in ROS levels relative to the group treated solely with polymyxin E (**Figures 1A, B**). Based on this premise, we propose that the antagonistic effect of nicorandil on the bactericidal mechanism of polymyxin E is predominantly attributable to its inhibition of the oxidative stress response elicited by polymyxin E.

Catalase and superoxide dismutase play pivotal roles in regulating cellular responses to oxidative stress (An et al., 2011). We investigated the impact of polymyxin E, with and without nicorandil, on the expression of *sodA* and *sodC* (superoxide dismutases) and *katE* and *katG* (catalase). We conducted RT-qPCR assays on GN 191035 after a 4-hour treatment with nicorandil, polymyxin E, or their combination. The study demonstrated that co-administration of nicorandil and polymyxin E significantly decreased the expression levels of *sodA*, *sodC*, *katE*, and *katG* compared to treatment with polymyxin E alone (**Figure 1C**). To corroborate these findings, we conducted a qPCR assay utilizing an alternative *K. pneumoniae* strain, GN 230444. Consistent with the observations in strain 191035, the expression levels of *sodA*, *sodC*, *katE*, and *katG* in GN 230444 were reduced following treatment with exogenous nicorandil (**Supplementary Figure S7**). The observed downregulation of SOD and KAT expression in the presence of nicorandil suggests that nicorandil mitigated the levels of ROS induced by polymyxin E.

### 3.4 Nicorandil released nitric oxide, which protects *K. pneumoniae* against polymyxin E

Research indicates that NO provides immediate protection against oxidative stress caused by hydrogen peroxide (H_2_O_2_) (Gusarov et al., 2009). Nicorandil, which contains a nitrate group (–ONO_2_) in its molecular structure, can release NO into the human body (**Figure 2A**). This study investigated the potential of nicorandil in releasing NO from bacterial systems. The accumulation of reactive nitrogen species (RNS) in the presence of nicorandil was evaluated using DAF-FM DA. The results showed a significant increase in intracellular RNS levels after nicorandil treatment (**Figures 2B, C**). Furthermore, no changes were observed when two structural analogues lacking the nitrate group, N-ethyl nicotinamide and N-(2-hydroxyethyl) nicotinamide, were introduced into the cultures (**Figures 2B, C**). Collectively, these findings suggested that nicorandil released NO via its nitrate groups.

To determine whether nicorandil mitigates the bactericidal effects of polymyxin E on *K. pneumoniae* through NO release, we evaluated the effects of N-ethyl nicotinamide, N-(2-hydroxyethyl) nicotinamide, and nicorandil, both independently and in combination with polymyxin E, on bacterial proliferation. This was achieved using spot dilution assays (**Figure 2D**) and growth curve inhibition analyses (**Figure 2E**).

The spot dilution assay indicated that combining polymyxin E with nicorandil significantly accelerated bacterial growth, showing an increase of 1 log_10_ CFU/mL at 4 hours and 3 log_10_ CFU/mL at 6 hours (**Figure 2D**). Conversely, no significant bacterial growth was detected when polymyxin E was combined with the two analogues or administered alone (**Figure 2D**). Polymyxin E exhibited effective bactericidal activity in growth curve inhibition analyses, regardless of the presence of N-ethyl nicotinamide and N-(2-hydroxyethyl) nicotinamide (**Figure 2E**). However, the addition of nicorandil significantly reduced this bactericidal effect (**Figure 2E**). This observation indicates that neither analogue replicates this effect, suggesting that the nitrate moiety is crucial for the antagonistic action of nicorandil. Subsequently, we conducted a comprehensive investigation into the temporal variations in NO levels released by nicorandil in bacterial systems, employing DAF-FM DA as the detection method. We further evaluated their impact on the efficacy of polymyxin E using a rapid killing assay. Our findings indicate that incubation with nicorandil for one hour prior to the addition of polymyxin E resulted in increased intracellular reactive nitrogen species (RNS) accumulation (**Supplementary Figure S8A**) and decreased bacterial clearance (**Supplementary Figure S8B**). Conversely, when incubation with nicorandil was extended to six hours before the addition of polymyxin E, the results were reversed (**Supplementary Figures S8C, D**). Collectively, these findings indicate that nicorandil releases NO, which confers protection to *K. pneumoniae* against Polymyxin E.

### 3.5 Nicorandil reduces the antimicrobial efficacy of polymyxin E in *in vivo* models

To evaluate nicorandil’s impact on polymyxin E’s antibacterial efficacy *in vivo*, researchers developed a mouse model of *K. pneumoniae*-induced lung injury and an infection model using *Galleria mellonella* larvae. In the murine lung model with *K. pneumoniae* GN 191035 (**Figure 3A**), polymyxin E treatment alone showed no statistically significant difference from the control group. The cohort receiving combined polymyxin E and nicorandil treatment exhibited a significantly higher pulmonary bacterial load than the cohort treated with polymyxin E alone (**Figure 3D**). Furthermore, lung pathology was evaluated, and semiquantitative scores were assigned to post-*K. pneumoniae* infection. Histopathological analysis revealed that all infected mice showed severe pneumonia, characterized by bronchitis, interstitial inflammation, edema, endothelialitis, pleuritis, and thrombus formation (**Figures 3B, C**).

We further evaluated our combinatorial approach *in vivo* using the *G. mellonella* infection model and performed statistical analysis of the survival curves. In the *G. mellonella* model infected with *K. pneumoniae* GN 191035, treatment with polymyxin E alone resulted in a 68.65% survival rate at 72 hours post-inoculation. In contrast, the mortality rate due to bacterial infection was significantly higher in *G. mellonella* specimens treated with the combination of nicorandil and polymyxin E, as illustrated in **Figure 3E**.

## 4 Discussion

The rise of multidrug-resistant bacterial infections significantly threatens public health, requiring the investigation of new therapeutic approaches (Abbas et al., 2024). The use of NO donors has garnered attention as a promising approach to enhance antibiotic efficacy (Zemke et al., 2014). However, our findings reveal a previously underappreciated aspect of this strategy, namely the potential antagonistic interactions between NO donors and certain antibiotics. This research examines the clinically important pathogen, carbapenem-resistant multidrug-resistant *K. pneumoniae*, and its resistance to polymyxin E, a critical last-resort treatment for multidrug-resistant Gram-negative bacterial infections. The increasing prevalence of resistance to polymyxin E underscores the urgency of understanding factors that may further compromise its efficacy (Liu et al., 2016; Tran et al., 2018). Our study explores the interaction between nicorandil, a commonly prescribed vasodilator with NO releasing properties, and polymyxin E, revealing potential antagonistic effects that could impact clinical treatment strategies.

Prior studies have shown that NO can boost antibiotic effectiveness, and combining NO donors with antibiotics can eliminate drug-resistant strains (van Sorge et al., 2013). Nonetheless, our findings reveal that nicorandil exhibits antagonistic interactions with polymyxin E in 51.6% of the tested *K. pneumoniae* clinical isolates, leading to a significant increase in bacterial proliferation. The antimicrobial efficacy of Polymyxin E against *K. pneumoniae* can be augmented by Gallium nitrate through the induction of ROS accumulation (Guo et al., 2023). Prior studies have shown that NO can improve bacterial resistance to antibiotics by reducing oxidative stress (Gusarov and Nudler, 2005, 2009). Our study corroborates this by showing that nicorandil significantly decreases ROS levels induced by Polymyxin E, as demonstrated by flow cytometric analysis and the downregulation of antioxidant enzyme genes, including *sodA*, *sodC*, *katE*, and *katG*. Therefore, it is hypothesized that NO may protect bacteria by alleviating the oxidative stress caused by Polymyxin E.

To further validate our findings, we employed both a murine lung injury model and a *Galleria mellonella* infection model. In the mouse model, the combination of nicorandil and polymyxin E significantly increased pulmonary bacterial load compared to polymyxin E alone, despite the lack of a significant difference between polymyxin E treatment and the control group. Histopathological analysis revealed severe pneumonia in all infected mice, with more pronounced pathology in those treated with the combination. Similarly, in the *Galleria mellonella* model, the combination of nicorandil and polymyxin E resulted in higher mortality compared to polymyxin E alone. These *in vivo* results corroborate our *in vitro* observations, confirming the antagonistic interaction between nicorandil and polymyxin E.

This suggests that nicorandil’s antagonistic effect on polymyxin E is primarily due to its ability to mitigate oxidative stress. Similar mechanisms have been observed with other antioxidants, such as doxofylline (Chen et al., 2021) and curcumin (Marathe et al., 2013). The study underscores the potential risk associated with combining nicorandil and polymyxin E, especially when treating infections caused by multidrug-resistant *K. pneumoniae*. Given the widespread use of nicorandil for ischemic heart disease, these findings have significant clinical implications for optimizing antimicrobial therapy and minimizing drug interactions. Future research should examine the interactions between nicorandil and other antimicrobial agents, and investigate methods to modulate NO release or increase oxidative stress to enhance polymyxin E efficacy. Further studies are also required to validate these findings across diverse clinical isolates and infection models.

## Data Availability

The datasets presented in this study can be found in online repositories. The names of the repository/repositories and accession number(s) can be found in the article/**Supplementary Material**.
