# Supplementary material for: Nicorandil reduces the antimicrobial effectiveness of polymyxin E against Klebsiella pneumoniae by decreasing reactive oxygen species accumulation
Source: Front Cell Infect Microbiol. 2025 Sep 17;15:1658194. doi: 10.3389/fcimb.2025.1658194 (PMC12484039; doi:10.3389/fcimb.2025.1658194)
Supplement: Supplementary file 1 [file DataSheet1.pdf]

Table S1. Antimicrobial Susceptibility for the selected clinical Strains

bacterial isolate	Lipopeptides	Carbapenems		Cephalosporins alkene		Monocyclic lactam	Tetracyclines		Quinolones		β -lactam compound
	PolE	IMP	MEM	FEP	CAZ	ATM	DOX	MNO	CIP	LVX	TZP
GN 200043	0.5	>32	>32	64	64	>128	16	8	>4	>8	>128
GN 200774	0.5	>32	>32	128	>128	128	>16	>16	>4	>8	>128
GN 200999	1	>32	>32	128	64	>128	>16	16	>4	>8	>128
GN 201286	0.5	>32	>32	64	32	>128	>16	16	>4	>8	>128
GN 190808	0.5	>32	>32	>128	>128	>128	16	8	>4	>8	>128
GN 191324	0.5	>32	>32	>128	>128	>128	>16	8	>4	>8	>128
GN 192105	1	>32	16	32	64	>128	>16	>16	>4	>8	>128
GN 181719	0.5	>32	>32	>128	128	>128	>16	16	>4	>8	>128
GN 182201	32	>32	>32	>128	>128	>128	>16	>16	>4	>8	>128
GN 172646	1	>32	>32	>128	64	>128	8	8	>4	>8	>128
GN 172769	1	>32	>32	>128	>128	>128	>16	8	>4	>8	>128

GN 172859	0.5	>32	>32	>128	>128	>128	>16	16	>4	>8	>128
GN 191035	64	>32	>32	32	64	64	>16	>16	>4	>8	>128
GN 230444	32	32	32	64	128	128	>16	>16	>4	>8	>128
GN 230445	32	32	32	64	128	128	>16	>16	>4	>8	>128
GN 230446	32	32	32	64	128	128	>16	>16	>4	>8	>128
GN 230447	32	32	32	64	128	128	>16	>16	>4	>8	>128
GN 230454	1	32	32	64	128	128	4	8	>4	>8	>128
GN 230455	1	32	32	64	32	128	>16	8	>4	>8	>128
GN 230456	0.5	32	32	64	128	128	>16	>16	>4	>8	>128
GN 230457	1	32	32	64	128	128	2	4	>4	>8	>128
GN 230458	1	32	32	64	128	128	>16	>16	>4	>8	>128
GN 230459	0.5	32	32	64	128	128	4	8	>4	>8	>128
GN 230460	0.5	32	32	64	128	128	>16	8	>4	>8	>128

GN 230461	0.5	32	32	64	128	128	4	8	>4	>8	>128
GN 230462	1	32	32	64	128	128	4	4	>4	>8	>128
GN 230463	1	32	32	64	128	128	>16	>16	>4	>8	>128
GN 230464	1	32	32	64	128	128	4	8	>4	>8	>128
GN 230466	1	32	32	64	128	128	>16	16	>4	>8	>128
GN 230467	0.5	32	32	64	128	128	>16	>16	>4	>8	>128
ATCC 43816	0.5	0.5	≤0.25	≤0.25	≤0.25	≤0.25	2	2	≤0.25	≤0.25	4

MIC, minimum inhibitory concentration. PolE, polymyxin E (the range of polymyxin E concentrations used in MIC test was 0.25 - 128 μg/mL); IMP, imipenem (0.25 - 32 μg/mL); MEM, meropenem (0.25 - 32μg/mL); FEP, cefepime (0.25 - 128 μg/mL); CAZ, ceftazidime (0.25 - 128 μg/mL); ATM, aztreonam (0.25 - 128 μg/mL); DOX, doxycycline (0.25 - 16 μg/mL); MNO, minocycline (0.25 - 16 μg/mL); CIP, ciprofloxacin (0.25 - 8 μg/mL); LVX, levofloxacin (0.25 - 8 μg/mL); TZP, piperacillin-tazobactam (1 - 128 μg/mL).

Table S2. Primers used in this study

Gene	Primer	Sequence 5` - 3`
sodA	*sodA*/F	CCGCTGAAGAGCTGATTACC
	sodA/R	TTGAAGTTCTCCACGGAACC
sodC	*sodC*/F	ATTACGATCCGCAGCATACC
	sodC/R	GACTGTCGGCCATGTTATCC
KatE	*KatE/F*	CGCATTGCTGACGATCAGAA
	KatE/R	ATCGGCTTTAGTCAACGCAG
KatG	*KatG/F*	CTTCATCGTCCATCGCCATA
	KatG/R	CGATGAGAAAGAGTGGCTGG
rrsE6	*rrsE6/F*	TTGACGTTACCCGCAGAAGAA
	rrsE6/R	GCTTGCACCCTCCGTATTACC

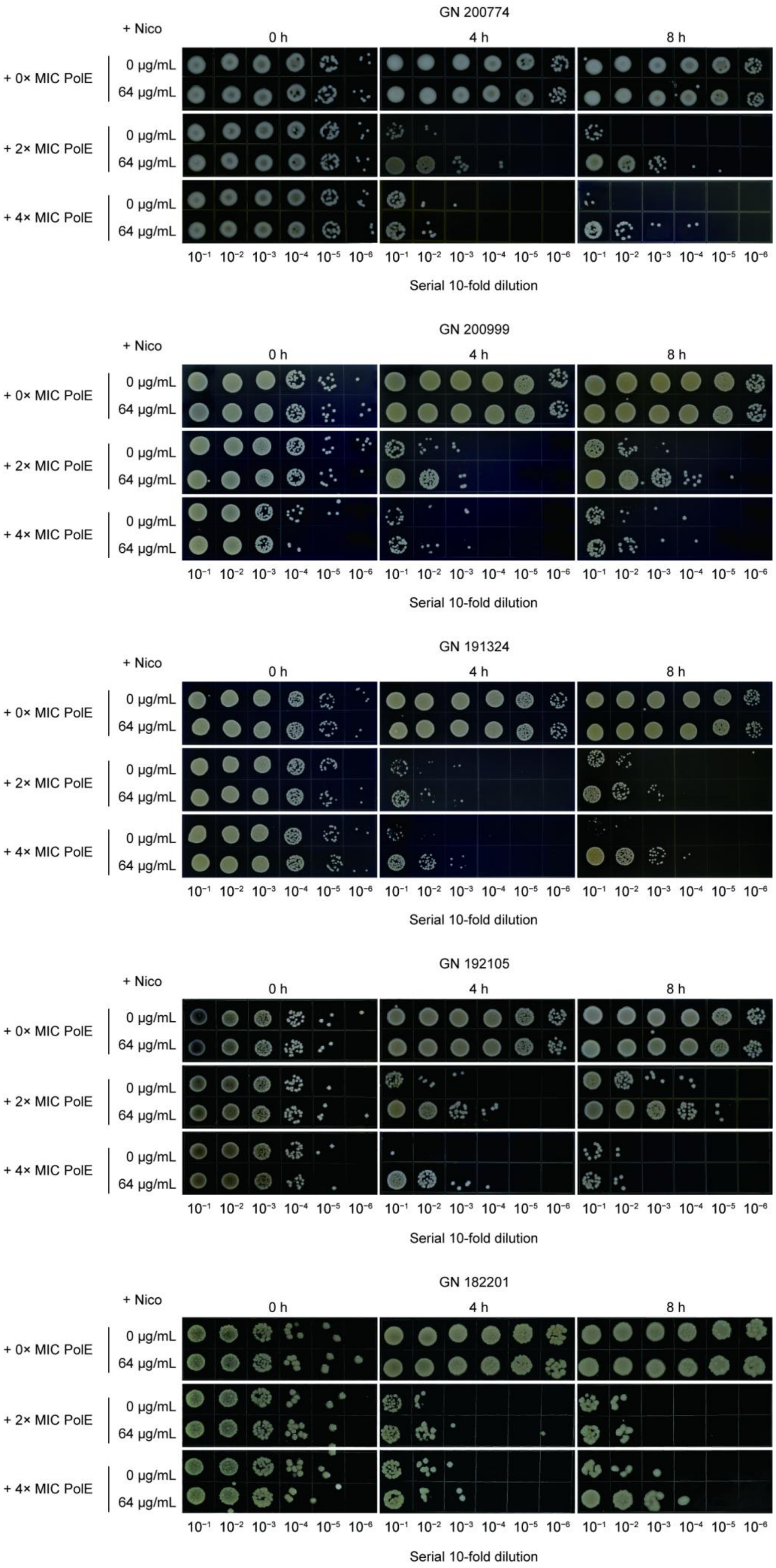
GN 200774
+ Nico
0 h
4 h
8 h
+ 0× MIC PolE
+ 2× MIC PolE
+ 4× MIC PolE
0 μg/mL
64 μg/mL
10⁻¹ 10⁻² 10⁻³ 10⁻⁴ 10⁻⁵ 10⁻⁶
Serial 10-fold dilution
GN 200999
GN 191324
GN 192105
GN 182201

Figure S1. Rapid killing assay using *Klebsiella pneumoniae* strains GN 200774, GN 200999,GN 191324, GN 192105, GN 182201. *K. pneumoniae* GN 200774, GN 200999, GN 191324, GN 192105, GN 182201 were untreated (Control) or treated with 2 × MIC and 4 × MIC polymyxin E (PolE), 64 μg/mL nicorandil (Nico), 2 × MIC and 4 × MIC polymyxin E and 64 μg/mL nicorandil (PolE&Nico). Serial 10-fold dilutions of samples were spotted onto MHA plates in 4-hour and 8-hour. All plates were incubated at 37 °C for 12 h and photographed. The images are representative of three independent replicate experiment.

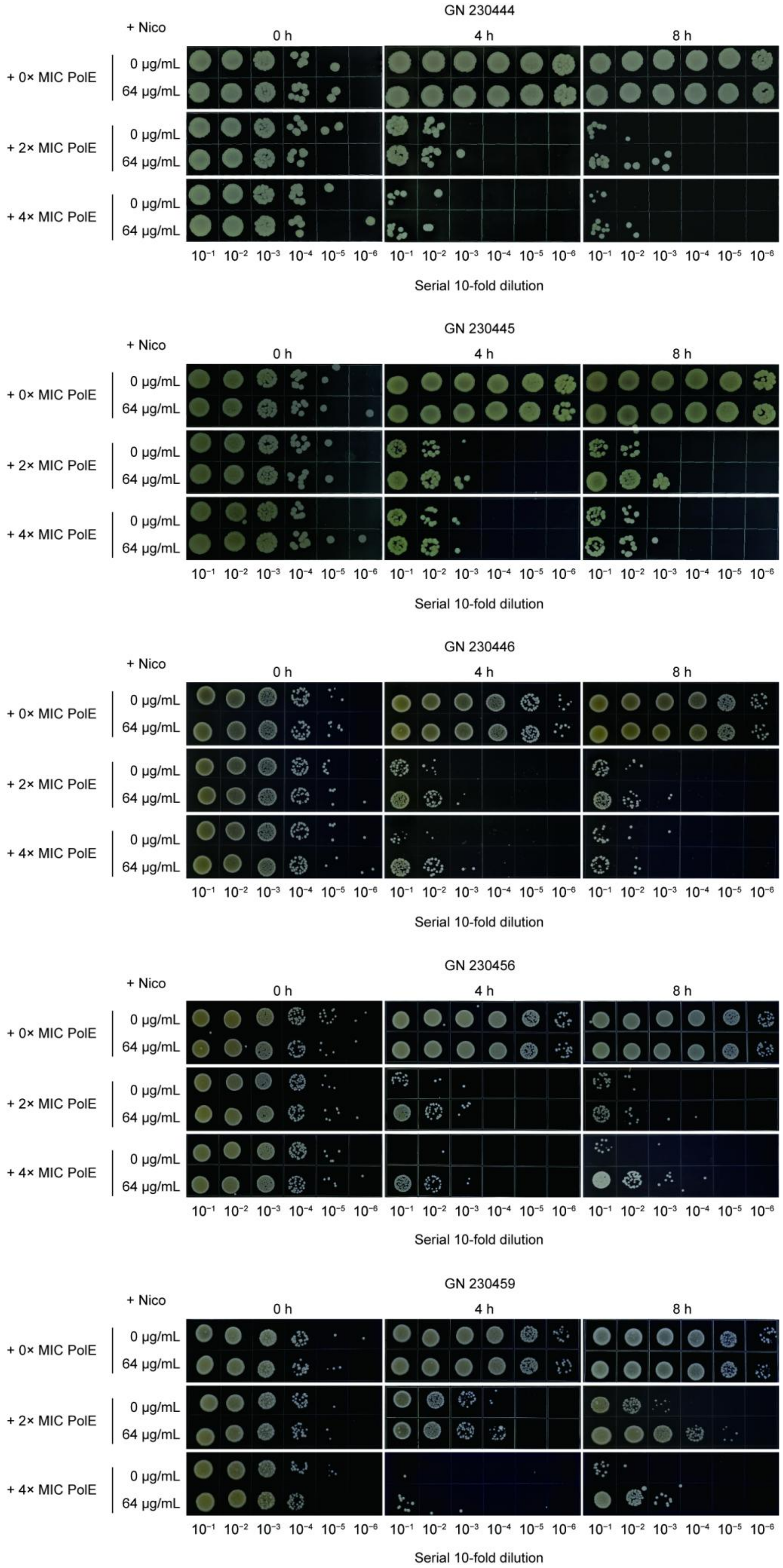
GN 230444
+ Nico
0 h
4 h
8 h
+ 0× MIC PolE
+ 2× MIC PolE
+ 4× MIC PolE
0 μg/mL
64 μg/mL
10⁻¹ 10⁻² 10⁻³ 10⁻⁴ 10⁻⁵ 10⁻⁶
Serial 10-fold dilution
GN 230445
+ Nico
0 h
4 h
8 h
+ 0× MIC PolE
+ 2× MIC PolE
+ 4× MIC PolE
0 μg/mL
64 μg/mL
10⁻¹ 10⁻² 10⁻³ 10⁻⁴ 10⁻⁵ 10⁻⁶
Serial 10-fold dilution
GN 230446
+ Nico
0 h
4 h
8 h
+ 0× MIC PolE
+ 2× MIC PolE
+ 4× MIC PolE
0 μg/mL
64 μg/mL
10⁻¹ 10⁻² 10⁻³ 10⁻⁴ 10⁻⁵ 10⁻⁶
Serial 10-fold dilution
GN 230456
+ Nico
0 h
4 h
8 h
+ 0× MIC PolE
+ 2× MIC PolE
+ 4× MIC PolE
0 μg/mL
64 μg/mL
10⁻¹ 10⁻² 10⁻³ 10⁻⁴ 10⁻⁵ 10⁻⁶
Serial 10-fold dilution
GN 230459
+ Nico
0 h
4 h
8 h
+ 0× MIC PolE
+ 2× MIC PolE
+ 4× MIC PolE
0 μg/mL
64 μg/mL
10⁻¹ 10⁻² 10⁻³ 10⁻⁴ 10⁻⁵ 10⁻⁶
Serial 10-fold dilution

Figure S2. Rapid killing assay using *Klebsiella pneumoniae* strains GN 230444, GN 230445, GN 230446, GN 230456, GN 230459. *K. pneumoniae* GN 200774, GN 200999, GN 191324, GN 192105, GN 182201, GN 230444, GN 230445, GN 230446, GN 230456, GN 230459 were untreated (Control) or treated with 2 × MIC and 4 × MIC polymyxin E (PolE), 64 μg/mL nicorandil (Nico), 2 × MIC and 4 × MIC polymyxin E and 64 μg/mL nicorandil (PolE&Nico). Serial 10-fold dilutions of samples were spotted onto MHA plates in 4-hour and 8-hour. All plates were incubated at 37 °C for 12 h and photographed. The images are representative of three independent replicate experiment.

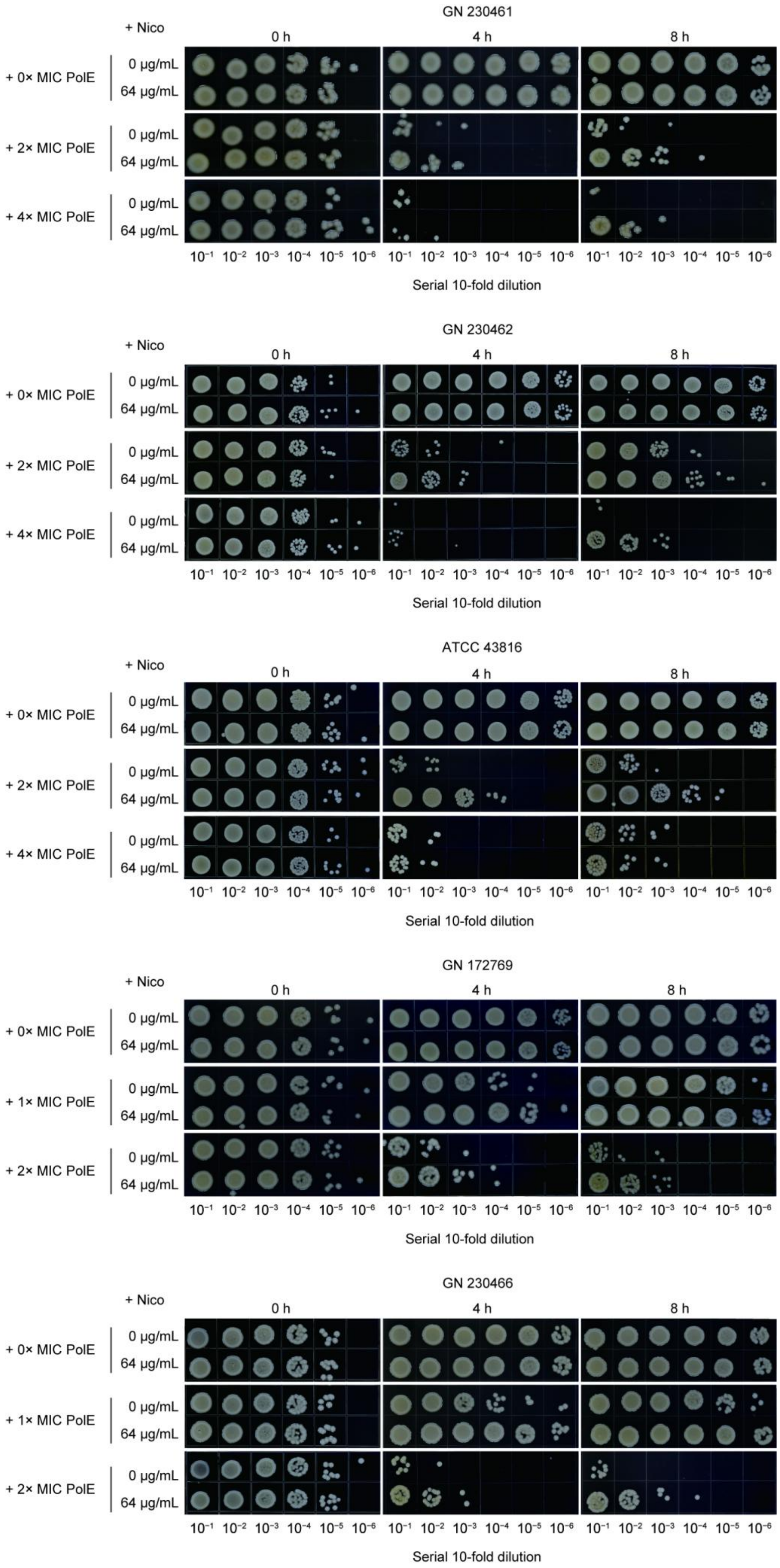
GN 230461
+ Nico
0 h
4 h
8 h
+ 0× MIC PolE
0 μg/mL
64 μg/mL
+ 2× MIC PolE
0 μg/mL
64 μg/mL
+ 4× MIC PolE
0 μg/mL
64 μg/mL
10⁻¹ 10⁻² 10⁻³ 10⁻⁴ 10⁻⁵ 10⁻⁶ 10⁻¹ 10⁻² 10⁻³ 10⁻⁴ 10⁻⁵ 10⁻⁶ 10⁻¹ 10⁻² 10⁻³ 10⁻⁴ 10⁻⁵ 10⁻⁶
Serial 10-fold dilution
GN 230462
+ Nico
0 h
4 h
8 h
+ 0× MIC PolE
0 μg/mL
64 μg/mL
+ 2× MIC PolE
0 μg/mL
64 μg/mL
+ 4× MIC PolE
0 μg/mL
64 μg/mL
10⁻¹ 10⁻² 10⁻³ 10⁻⁴ 10⁻⁵ 10⁻⁶ 10⁻¹ 10⁻² 10⁻³ 10⁻⁴ 10⁻⁵ 10⁻⁶ 10⁻¹ 10⁻² 10⁻³ 10⁻⁴ 10⁻⁵ 10⁻⁶
Serial 10-fold dilution
ATCC 43816
+ Nico
0 h
4 h
8 h
+ 0× MIC PolE
0 μg/mL
64 μg/mL
+ 2× MIC PolE
0 μg/mL
64 μg/mL
+ 4× MIC PolE
0 μg/mL
64 μg/mL
10⁻¹ 10⁻² 10⁻³ 10⁻⁴ 10⁻⁵ 10⁻⁶ 10⁻¹ 10⁻² 10⁻³ 10⁻⁴ 10⁻⁵ 10⁻⁶ 10⁻¹ 10⁻² 10⁻³ 10⁻⁴ 10⁻⁵ 10⁻⁶
Serial 10-fold dilution
GN 172769
+ Nico
0 h
4 h
8 h
+ 0× MIC PolE
0 μg/mL
64 μg/mL
+ 1× MIC PolE
0 μg/mL
64 μg/mL
+ 2× MIC PolE
0 μg/mL
64 μg/mL
10⁻¹ 10⁻² 10⁻³ 10⁻⁴ 10⁻⁵ 10⁻⁶ 10⁻¹ 10⁻² 10⁻³ 10⁻⁴ 10⁻⁵ 10⁻⁶ 10⁻¹ 10⁻² 10⁻³ 10⁻⁴ 10⁻⁵ 10⁻⁶
Serial 10-fold dilution
GN 230466
+ Nico
0 h
4 h
8 h
+ 0× MIC PolE
0 μg/mL
64 μg/mL
+ 1× MIC PolE
0 μg/mL
64 μg/mL
+ 2× MIC PolE
0 μg/mL
64 μg/mL
10⁻¹ 10⁻² 10⁻³ 10⁻⁴ 10⁻⁵ 10⁻⁶ 10⁻¹ 10⁻² 10⁻³ 10⁻⁴ 10⁻⁵ 10⁻⁶ 10⁻¹ 10⁻² 10⁻³ 10⁻⁴ 10⁻⁵ 10⁻⁶
Serial 10-fold dilution

Figure S3. Rapid killing assay using *Klebsiella pneumoniae* strains GN 230461, GN 230462, ATCC 43816，GN 172769, GN 230466. *K. pneumoniae* GN 230461, GN 230462, ATCC 43816 were untreated (Control) or treated with 2 × MIC and 4 × MIC polymyxin E (PolE), 64 μg/mL nicorandil (Nico), 2 × MIC and 4 × MIC polymyxin E and 64 μg/mL nicorandil (PolE&Nico). Serial 10-fold dilutions of samples were spotted onto MHA plates in 4-hour and 8-hour. *K. pneumoniae* GN 172769, GN 230466 were untreated (Control) or treated with 1 × MIC and 2 × MIC polymyxin E (PolE), 64 μg/mL nicorandil (Nico), 1 × MIC and 2 × MIC polymyxin E and 64 μg/mL nicorandil (PolE & Nico). Serial 10-fold dilutions of samples were spotted onto MHA plates in 4-hour and 8-hour. All plates were incubated at 37 °C for 12 h and photographed. The images are representative of three independent replicate experiments.

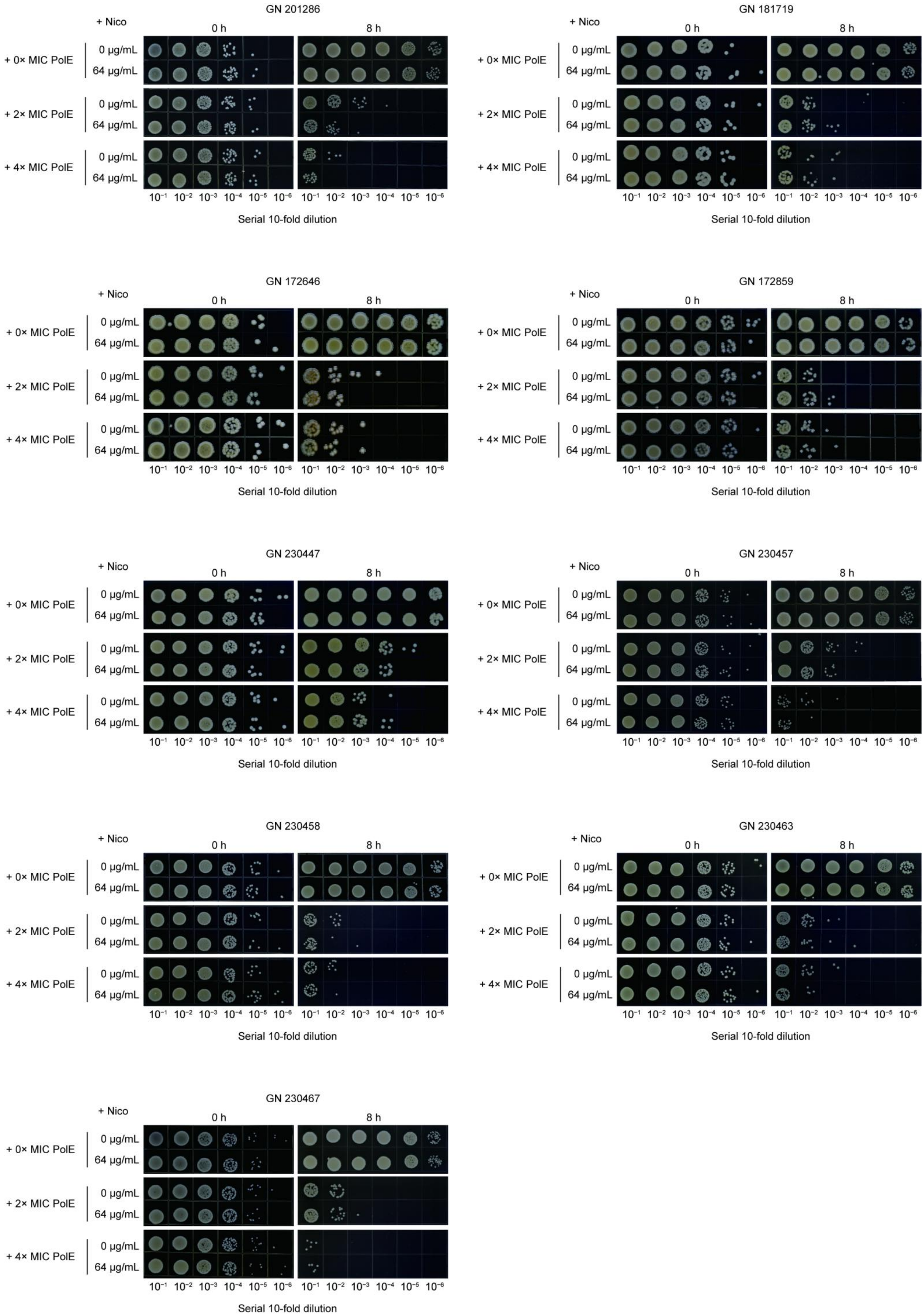
GN 201286
+ Nico
0 h
8 h
+ 0× MIC PolE
+ 2× MIC PolE
+ 4× MIC PolE
0 μg/mL
64 μg/mL
10^{-1} 10^{-2} 10^{-3} 10^{-4} 10^{-5} 10^{-6}
Serial 10-fold dilution
GN 181719
+ Nico
0 h
8 h
+ 0× MIC PolE
+ 2× MIC PolE
+ 4× MIC PolE
0 μg/mL
64 μg/mL
10^{-1} 10^{-2} 10^{-3} 10^{-4} 10^{-5} 10^{-6}
Serial 10-fold dilution
GN 172646
+ Nico
0 h
8 h
+ 0× MIC PolE
+ 2× MIC PolE
+ 4× MIC PolE
0 μg/mL
64 μg/mL
10^{-1} 10^{-2} 10^{-3} 10^{-4} 10^{-5} 10^{-6}
Serial 10-fold dilution
GN 172859
+ Nico
0 h
8 h
+ 0× MIC PolE
+ 2× MIC PolE
+ 4× MIC PolE
0 μg/mL
64 μg/mL
10^{-1} 10^{-2} 10^{-3} 10^{-4} 10^{-5} 10^{-6}
Serial 10-fold dilution
GN 230447
+ Nico
0 h
8 h
+ 0× MIC PolE
+ 2× MIC PolE
+ 4× MIC PolE
0 μg/mL
64 μg/mL
10^{-1} 10^{-2} 10^{-3} 10^{-4} 10^{-5} 10^{-6}
Serial 10-fold dilution
GN 230457
+ Nico
0 h
8 h
+ 0× MIC PolE
+ 2× MIC PolE
+ 4× MIC PolE
0 μg/mL
64 μg/mL
10^{-1} 10^{-2} 10^{-3} 10^{-4} 10^{-5} 10^{-6}
Serial 10-fold dilution
GN 230458
+ Nico
0 h
8 h
+ 0× MIC PolE
+ 2× MIC PolE
+ 4× MIC PolE
0 μg/mL
64 μg/mL
10^{-1} 10^{-2} 10^{-3} 10^{-4} 10^{-5} 10^{-6}
Serial 10-fold dilution
GN 230463
+ Nico
0 h
8 h
+ 0× MIC PolE
+ 2× MIC PolE
+ 4× MIC PolE
0 μg/mL
64 μg/mL
10^{-1} 10^{-2} 10^{-3} 10^{-4} 10^{-5} 10^{-6}
Serial 10-fold dilution
GN 230467
+ Nico
0 h
8 h
+ 0× MIC PolE
+ 2× MIC PolE
+ 4× MIC PolE
0 μg/mL
64 μg/mL
10^{-1} 10^{-2} 10^{-3} 10^{-4} 10^{-5} 10^{-6}
Serial 10-fold dilution

Figure S4. Rapid killing assay using *Klebsiella pneumoniae* strains GN 201286, GN 181719, GN 172646, GN 172859, GN 230447, GN 230457, GN 230458, GN 230463, GN 230467. *K. pneumoniae* GN 201286, GN 181719, GN 172646, GN 172859, GN 230447, GN 230458, GN 230463, GN 230467 were untreated (Control) or treated with 2 × MIC and 4 × MIC polymyxin E (PolE), 64 μg/mL nicorandil (Nico), 2 × MIC and 4 × MIC polymyxin E and 64 μg/mL nicorandil (PolE&Nico). Serial 10-fold dilutions of samples were spotted onto MHA plates in 4-hour and 8-hour. All plates were incubated at 37 °C for 12 h and photographed. The images are representative of three independent replicate experiments.

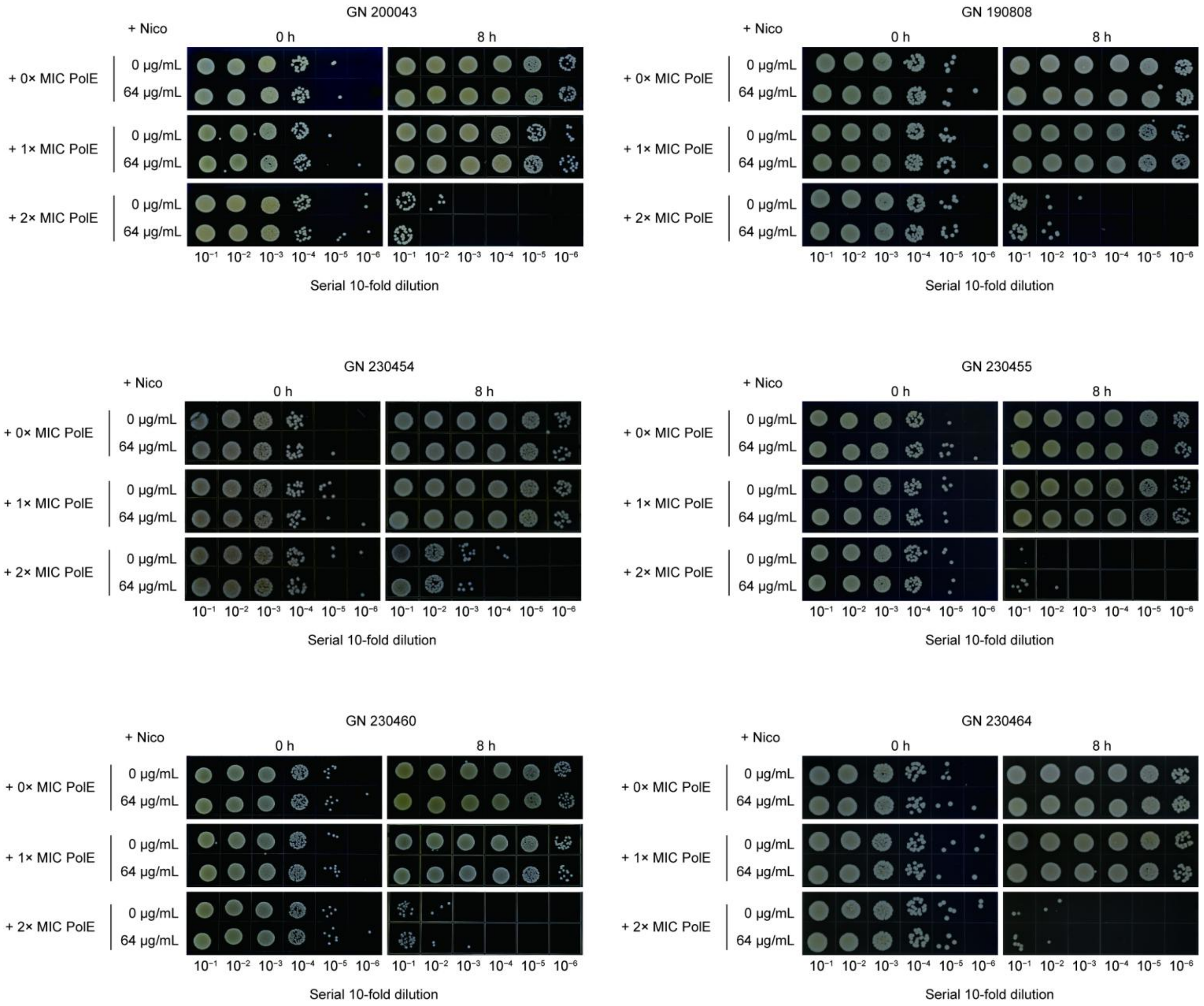

Figure S5. Rapid killing assay using *Klebsiella pneumoniae* strains GN 200043, GN 190808, GN 230454, GN 230455, GN 230460, GN 230464 *K. pneumoniae* GN 200043, GN 190808, GN 230454, GN 230455, GN 230457, GN 230460, GN 230464 were untreated (Control) or treated with 1 × MIC and 2 × MIC polymyxin E (PolE), 64 μg/mL nicorandil (Nico), 1 × MIC and 2 × MIC polymyxin E and 64 μg/mL nicorandil (PolE&Nico). Serial 10-fold dilutions of samples were spotted onto MHA plates in 4-hour and 8-hour. All plates were incubated at 37 °C for 12 h and photographed. The images are representative of three independent replicate experiments.

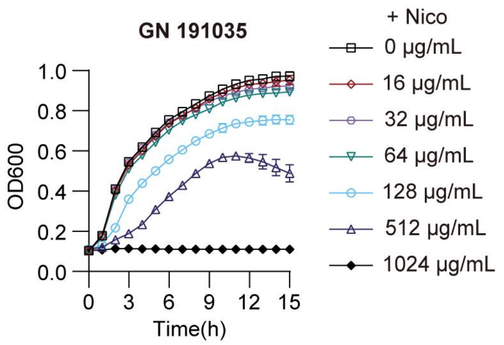

Figure S6. The effect of Nicorandil on the growth of *K. pneumoniae*. The growth curve of GN 191035 cultured in MHB supplemented with or without the indicated concentration of nicorandil. The data from n = 4 biological replicates are reported as the mean ± SD.

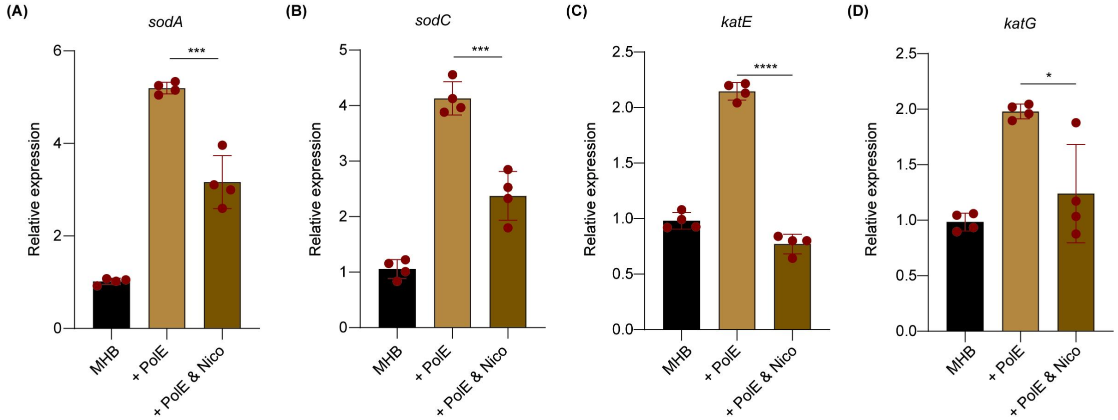

Figure S7. Real-time quantitative PCR was employed in the analysis of strain GN 230444. The mRNA expression levels of *sodA* **(A)**, *sodC* **(B)**, *katE* **(C)**, and *katG* **(D)** were evaluated in the control group (MHB), the polymyxin E-only group (+ PolE), and the group treated with a combination of polymyxin E and nicorandil (+ PolE & Nico). Experiments were conducted in 50 ml Eppendorf tubes with treatments of nicorandil (64 μg/mL), 1/2 MIC polymyxin E (16 μg/mL), and their combination. The mRNA expression levels were normalized relative to *rrsE6*. Data are expressed as mean ± standard deviation (n = 4).

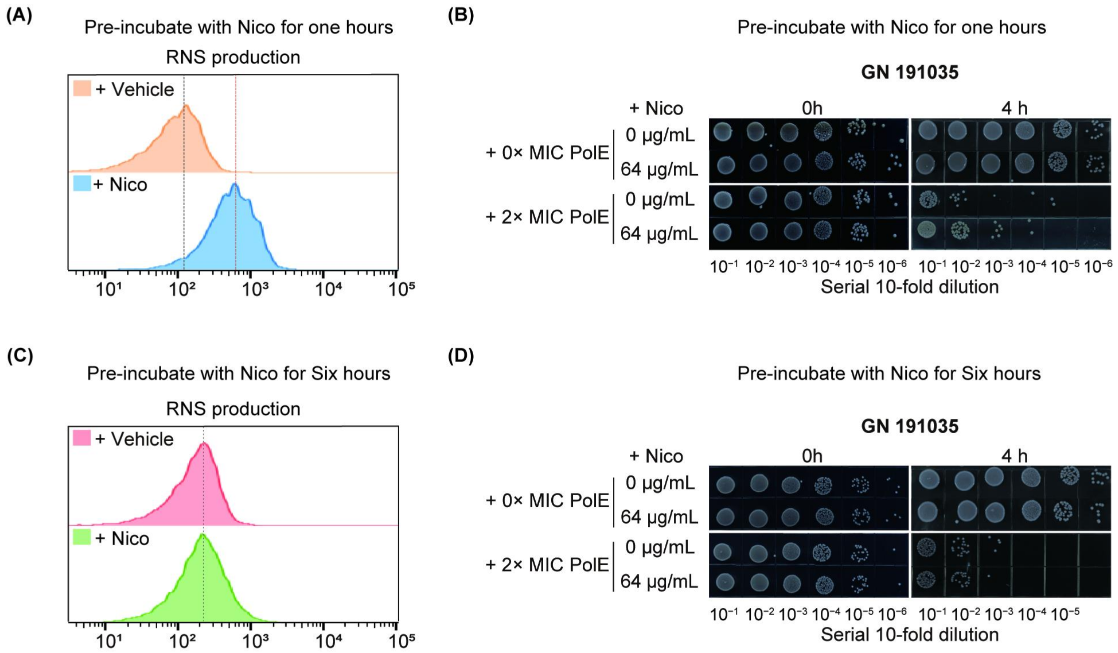

Figure S8. The release of NO by nicorandil and its impact on the protection of bacterial cells. (A and C) The generation of reactive nitrogen species (RNS) in samples subjected to nicorandil exposure for one hour **(A)** and six hours **(C)** was evaluated through flow cytometry to quantify RNS levels. **(B and D)** Rapid killing assay. The influence of bacterial RNS levels in samples exposed to nicorandil for for one hour **(B)** and six hours **(D)** on the efficacy of polymyxin E was examined.
